# Prismatic Adaptation in the Rehabilitation of Neglect Patients: Does the Specific Procedure Matter?

**DOI:** 10.3389/fnhum.2013.00137

**Published:** 2013-04-09

**Authors:** Alessio Facchin, Roberta Daini, Alessio Toraldo

**Affiliations:** ^1^Department of Brain and Behavioral Sciences, University of PaviaPavia, Italy; ^2^Department of Psychology, University of Milano-BicoccaMilano, Italy

In rehabilitation studies, adaptation to lateral displacing prisms (rightward optical deviation) has been shown to reduce many manifestations of unilateral spatial neglect (USN) (Rossetti et al., 1998; Striemer and Danckert, 2010) and, when compared to other bottom-up techniques, has been proved to be effective for a longer time (Luaute et al., 2006a; Pisella et al., 2006; Newport and Schenk, 2012).

Prism adaptation (PA) itself is not a new technique; it has been used as a tool to investigate perceptual and motor control and adaptation for over a century (Helmholtz, 1910/1924; Held and Hein, 1958), but it has been used in neuropsychological rehabilitation only recently (Rossetti et al., 1998). PA is particularly suited to both theoretical and applied research because it produces observable effects in a short time (Redding and Wallace, 1997; Michel et al., 2003; Michel, 2006; Bultitude and Woods, 2010).

According to a standard procedure (Redding and Wallace, 1997, 2002) when a subject performs a pointing task through displacing prism lenses, his/her initial movements typically show an error in the direction of the prismatic shift. After a few trials such an error disappears and adaptation occurs. After this adaptation process, when prism goggles are removed, a pointing error appears in the opposite direction. This phenomenon is called aftereffect (AE), and is the hallmark of PA. In order to minimize the decay of the AE, an Open loop pointing (OLP) procedure, without visual feedback from the hand, can be used both before and after PA. The difference between these two sessions is considered to be the best measure of AE.

The most widespread PA procedure is the following. The subject is seated in front of a table, with his/her chin on a chinrest to prevent head movements. The subject has to perform fast hand movements starting from a fixed position on the table, near the body midline, to one or more targets that also lie on the table, within reaching distance. The starting position of the hand is usually occluded, so subjects can only see their own hand during the final part of the movement (Redding et al., 2005). This procedure, called terminal exposure, cannot be used as a rehabilitation technique with most neglect patients, because they frequently have left hemiplegia that reduces their global mobility. Hence different PA procedures were proposed specifically for rehabilitating neglect patients. In one popular procedure patients are required to perform simple pointing movements from the sternum to two or more landmarks placed on a table or on a tilted board, within reaching distance. Since the whole of the arm is visible during the movement, this procedure has been called concurrent exposure (Cohen, 1967). It is easy to perform for most neglect patients, but it does not allow one to assess the adaptation process directly because online corrections of the movement make pointing errors disappear. Moreover AE assessment is difficult; the first study of PA in neglect (Rossetti et al., 1998) used pointing to the subjective “straight ahead” (SSA) as a measure of AE.

Open loop pointing estimates of AE can be obtained with terminal exposure procedures in neglect patients using a wooden box to prevent visual feedback. The box is open on both the side facing the patient and the opposite side facing the experimenter who presents stimuli in different positions (Frassinetti et al., 2002). The box allows the patient to see only the final part of the movement, the gap between the box and the patient's trunk being covered with a black curtain; this apparatus allows one to obtain both online adaptation and final AE measures directly and precisely with a single setup. Usually, patients are required to point with their (right) index finger from the sternum to one of three targets placed at −20 or +20° from the midline. Some variations of this procedure have been proposed (Fortis et al., 2010; Wilms and Mala, 2010). The procedure with the PA Box tends to be rather long because both PA and AE measures are taken with the same apparatus, and movements are perceived as unnatural. Hence, overall, the technique is perceived as boring by the patients.

Recently a more patient friendly task has been proposed: PA was applied with ecological visuo-motor (VM) activities (Shiraishi et al., 2008; Fortis et al., 2010). Patients performed different VM activities consisting of manipulating common objects while wearing prismatic goggles. To estimate AE, OLP was administered before and after PA, using a PA box (Fortis et al., 2010). Since there are no restrictions on the visual input coming from the arm, this PA procedure can be classified as a concurrent exposure technique.

Concurrent and terminal exposure procedures are very different in terms of the patient's experience, but are they equally effective in neglect rehabilitation? Two studies tackled this issue, with opposite results (Fortis et al., 2010; Ladavas et al., 2011). The aim of the present work is to discuss the two methods and the aforementioned studies in the wider context of a general model of PA (Redding and Wallace, 1997, 2002, 2006).

Fortis et al. (2010) compared PA obtained with a terminal exposure procedure, and PA obtained using a VM task. They found that both procedures had similar rehabilitative efficacy. Not surprisingly, patients preferred to perform their rehabilitation by means of VM tasks. Ladavas et al. (2011) compared concurrent and terminal exposure by using a PA box with different amplitudes of visual feedback (terminal exposure: the last 12 cm of the movement were visible; concurrent exposure: the second half of the movement was visible). Terminal exposure produced larger rehabilitative effects than concurrent exposure. In both studies (Fortis et al., 2010; Ladavas et al., 2011) rehabilitative effects were measured by means of neuropsychological tests or batteries assessing neglect, which were administered before and after treatment (10 sessions of PA of about 20–30 min each). Clearly, the results of the two aforementioned researches are in contrast to each other. Indeed, VM tasks and pointing with concurrent exposure have in common a free-view of the arm and both allow the use of visual feedback from the movement. So we consider both VM tasks and concurrent exposure pointing as two procedures of concurrent exposure.

Both studies focused on the final rehabilitative effects – the impact on neglect measures – and failed to take into account the main factor that causes such effects, i.e., the adaptation process. Clearly if adaptation has been induced by using a PA box, one can derive a direct measure of adaptation – error reduction. Such a direct assessment is impossible if PA has been obtained by a VM task. So in the latter case, the only possibility of measuring the level of adaptation is an indirect one: by looking at the AE (Harris, 1963; Redding et al., 2005). Indeed AE magnitude is the same in patients and controls (Sarri et al., 2008; Facchin et al., in press). If different methods induce a comparable level of adaptation, an identical AE should be found. Previous studies comparing concurrent and terminal exposure procedures in healthy subjects found no significant difference in AE (Uhlarik and Canon, 1971; Choe and Welch, 1974; Redding and Wallace, 1988). Redding and Wallace (1993) measured AE several times during the PA process, and could detect some difference only at the beginning of the procedure, with an advantage of concurrent over terminal exposure. Such a difference later disappeared.

So, did the AE differ according to the procedure used in the two aforementioned studies? Both papers report a similar AE for both concurrent and terminal exposure. Both studies compared the two types of exposure within patients – thus providing a safer test of the hypothesis that they both produce the same AE. So, given that a similar AE was found with concurrent and terminal exposure in both studies, the inference can be made that the stimulation given by PA and the mechanism involved were the same in both cases. Why, then, did Ladavas et al. (2011) find a difference in neglect recovery? The question becomes, what is the relationship between AE (as assessed by OLP) and neglect recovery? Many studies did not find any clear relationship between AE and neglect recovery (Serino et al., 2007; Sarri et al., 2008; Ladavas et al., 2011), while others found a relationship only after having partialed out the effect of other explanatory variables (Fortis et al., 2010). The time scale discrepancy between the AE, which typically lasts for seconds or minutes, and neglect recovery, which can last for hours, days, or even weeks, has been well known since the beginning of research on PA in neglect rehabilitation (Frassinetti et al., 2002). AE confirms only that adaptation has occurred, but its size does not predict the improvement of neglect.

The dissociation between AE and rehabilitation efficacy has also been confirmed in anatomo-functional studies. The structures underlying PA seem to be intact in most neglect patients (left posterior parietal cortex, left superior temporal gyrus, right cerebellum) (Luaute et al., 2006b, 2009; Shiraishi et al., 2008) and this explains the occurrence of a normal adaptation process in this population. Other structures responsible for the mechanism of recovery induced by PA could be either injured (explaining cases where no improvement was found) or intact (significant improvement), independent of the areas involved in PA listed above.

We are left with the question of why different exposure procedures induce equal AE. This fact is a natural consequence of the mechanisms which have been assumed to underlie PA in an influential model. According to Redding and Wallace (Redding and Wallace, 1997, 2002; Redding et al., 2005), PA is due to two main processes: recalibration and realignment. The former is essentially a strategic cognitive process yielding a direct reduction of the error given by prisms; recalibration appears early in the procedure, as it requires a few trials to be triggered. The latter is a fully automatic reorganization of specific spatial maps (based on different frames of reference), and occurs later (and more slowly) in time. Realignment is defined as a kind of implicit perceptual learning (Redding and Wallace, 1997, 2002; Redding et al., 2005) and seems to be crucial in neglect rehabilitation (Redding and Wallace, 2006, 2009). To observe PA, recalibration (beginning after just a few trials) is not sufficient; a realignment of spatial maps is necessary, which can only be developed after several trials.

One crucial difference between concurrent and terminal exposure is in the amount of direct visual feedback from the pointing errors. In the terminal exposure condition, a large error is visible in the first trials, and the reduction of such an error in the following trials demonstrates that recalibration is indeed occurring. In the concurrent exposure condition, little or no error is visible from the first trials (because full visual feedback is available to correct the movement), so little recalibration takes place. By contrast, realignment, which is an automatic process, would develop across trials in both conditions in exactly the same way. More generally, Redding and Wallace (1997) assume that all methods of adaptation that use a visuo-manual task requiring precise movements toward a target, present an identical AE, exactly because PA in all of them depends on the same, automatic process of realignment.

According to this claim, the PA procedure should be selected on the basis of considerations other than its alleged “efficacy” (AE) – which, as we have shown, is expected to be identical in all procedures. Namely, it should be chosen taking into account the skills of the patient, his/her clinical conditions and needs. In the acute phase, or when patients have limited sustained attention, PA could be more easily performed via VM tasks or free-view pointing, perhaps toward center or right (if neglect prevents the patient from pointing leftwards). Terminal exposure might be an option only if a patient is able to perform it. With some patients the standard procedure involving AE estimates both before and after PA could be too long and demanding. Adaptation occurs in almost all patients, hence AE assessment could be given up, or, in the case of repeated sessions, could be administered only on the first session. If VM tasks are used, the optical aberration of prisms, i.e., chromatic aberration, distortion, and field curvature (Cotter, 2002; Facchin et al., in press) should be considered, as it can be more disturbing than with simple pointing. Whatever the choice, PA should consist of at least 90–100 trials (Frassinetti et al., 2002; Dijkerman et al., 2003), to be sure that realignment take place (see for negative results with less than 90 trials (Dijkerman et al., 2004; Keller et al., 2008).

In conclusion, the application of terminal exposure (pointing task) or concurrent exposure (pointing task or VM task) adaptation should be selected according to the patient's needs, because, from the point of view of adaptation “efficacy” they are likely to be equivalent. If neglect is moderate or severe, a PA box is very difficult to use and pointing in free-view is preferable. VM tasks, which are less boring, make it easier for the clinician to motivate patients to join the rehabilitation program. Furthermore, performing a set of easy daily activities would help the patient not only in terms of neglect improvement, but also as a form of general rehabilitation therapy.
